# Psychiatric medication prescriptions increasing for college students above and beyond the COVID-19 pandemic

**DOI:** 10.1038/s41598-023-46303-9

**Published:** 2023-11-04

**Authors:** Agustina M. Marconi, Ursula S. Myers, Bjorn Hanson, Sarah Nolan, Elena Beatriz Sarrouf

**Affiliations:** 1 University Health Services, University of Wisconsin Madison, 333 East Campus Mall, Madison, WI 53715 USA; 2 Medical University of South Carolina (MUSC), 171 Ashley Ave, Charleston, South Carolina 29425 USA; 3 Direction of Epidemiology, Province of Tucuman, Virgen de La Merced 196, San Miguel de Tucuman, Tucuman Argentina

**Keywords:** Health care, Public health

## Abstract

Psychiatric medication prescriptions for college students have been rising since 2007, with approximately 17% of college students prescribed medication for a mental health issue. This increase mirrors overall increases in both mental health diagnoses and treatment of university students. As psychiatric medication prescriptions for college students were increasing prior to pandemic, the goal of this study was to compare these prescriptions over the years, while accounting for the added stressor of the COVID-19 pandemic. This study utilized cross-sectional, retrospective data from a cohort of college students receiving care from the university’s health service. We examined prescriptions for mental healthcare from 2015 to 2021. There was a significant increase in the percentage of psychiatric medication prescriptions in 2020 (baseline 15.8%; threshold 3.5%) and 2021 (baseline 41.3%; threshold 26.3%) compared to the historical baseline average for the whole sample and as well as for female students (2020 baseline 21.3% and threshold 4.6%; 2021 baseline 55.1% and threshold 33.7%). Within these years, we found higher trends for prescriptions in April–May as well as September–December. Overall, we found that psychiatric medication prescriptions have continued to rise through the years, with a large increase occurring during the pandemic. In addition, we found that these increases reflect the academic year, which is important for university health centers to consider when they are planning to staff clinics and plan the best way to treat college students with mental health difficulties in the future.

## Introduction

Rates of psychiatric medication prescriptions for college students have been rising since 2007, with approximately 17% of college students prescribed medication for a mental health issue. Most of these prescriptions were antidepressants, anti-anxiety, and psychostimulants^1^. Anxiety disorders were the most prevalent followed by mood disorders, substance use disorders and behavioral disorders^2^. The increase in prescriptions mirrors overall increases in both mental health diagnoses as well as treatment in university students during the same period^3^. The life transitions occurring between 18–25 years old, such as leaving home for the first time, having to become an independent adult, as well as the majority of most mental health diagnoses presenting by age 25, are all likely causes for the need for mental health treatment for college age individuals^4, 5^. Several studies have also found that mental health diagnoses and prescriptions were more likely at certain times of the academic year. For example, appointments increase in September as the school year is starting and peak in December toward the time of final exams, and this cycle repeats during the spring semester^6^. On the other hand, untreated mental health diagnosis during college years was associated with worst academic outcomes, college withdrawal, and pauses in enrollment^7, 8^.

In addition to the stressors young adults face while in college, disasters are common triggers for increases in mental health disorders such as depressive disorders, anxiety disorders, posttraumatic stress disorder (PTSD), and substance use disorders^9^. Relatedly, there is often an increase in pharmacological interventions following a variety of disasters including earthquakes^10, 11^ terrorist attacks^12, 13^ cyclones^14^, and other disasters^15^. The most common increases in mental health prescriptions following disasters include antidepressants^10, 11^ and anxiety medications^13^. Most recently, the COVID-19 pandemic has had dramatic impacts on individuals’ mental health globally, with international studies reporting prescriptions for mental health disorders increased in 2020^16^. Within the United States, antidepressants and anxiolytic prescriptions increased when compared to historical data^17^. For college students, the physical distancing required to slow the spread of the disease led to closure of in person learning and shifting to virtual education in March 2020^18^. This abrupt shift in learning format as well as the enduring social isolation and COVID-related anxiety (e.g., fear of loved ones or themselves being ill or dying from COVID, work reductions, adapting to a new learning modality) negatively impacted students, with 48% of the students reporting moderate-to-severe levels of depression, 39% of the students reporting moderate-to-severe levels of anxiety, and 18% of students reporting suicidal thoughts 18%^19–21^. Studies comparing pre- and during-pandemic surveys report a significant increase in depression, eating disorders among others during pandemic for college students^22^.

As psychiatric medication prescriptions for college students were increasing prior to the pandemic, the goal of this study was to compare prescriptions over the years, while accounting for the added stressor of the COVID-19 pandemic. We hypothesized that prescriptions would continue to be higher, over and above what was seen during 2020, and these increased numbers would reflect the academic year, with higher prescriptions seen in December and May.

## Method

### Setting

This project was completed at UW-Madison, a large public university in the Midwest United States. The university had a fall term enrollment of 45,540 students in undergraduate, Master’s and doctoral degree programs. In 2020, 69.5% of enrolled students were undergraduates, 52.2% were female, 12.9% were international students, and 65% were white^23^. All students are eligible to be seen at the university health clinic (UHS) which includes mental health and medical services. Of students seen by a medical provider, 23% reported taking psychiatric medications in the 2017–2018 academic year^24^.

All experimental protocols were approved by UW-Madison Health Sciences and Minimal Risk Research Institutional Review Board (IRB). The UW-Madison Health Sciences and Minimal Risk Research Institutional Review Board (IRB) determined that the proposed activity is not research involving human subjects as defined by the Department of Health and Human Services (DHHS) and Food and Drug Administration (FDA) regulations (Submission ID number: 2021-1459). Therefore, and due to the numerical nature of the data, the UW-Madison Health Sciences and Minimal Risk Research Institutional Review Board (IRB) waived the need of informed consent. All methods in this study were performed in accordance with the relevant guidelines and regulations.

### Switch to virtual care

In the initial stages of the pandemic, it was evident that UHS had to strike a delicate balance between the potential dangers of undetected or untreated events and the imperative of minimizing risks to staff, patients, and the broader community that come with conducting traditional in-person visits during a pandemic. Prior to March 13, 2020, access to all UHS services operated normally. However, in response to the enduring global health crisis, the primary method of scheduling appointments, web-booking, was temporarily halted for both Medical and Mental Health. Instead, telephone visits conducted by a nurse or advanced practice provider were implemented as a means of triage^25^. Mental Health providers reassessed 12% of the students in individual counseling sessions to determine if they could continue to receive telehealth services or if they needed to connect with local resources. During the next months, Mental Health departments lost 5% clinical time due to furlough and though the services remained available with waitlists consistent with previous semesters.

Life on campus underwent a significant transformation during this particular period. Students received guidance to vacate the dormitories over spring break, and following their return, on March 23, 2020, all classes transitioned to virtual instruction exclusively. Throughout the summer, classes continued in an online format, with a blend of online and in-person classes for the majority of the fall semester. Towards the end of the fall semester in 2020, classes reverted to an online-only format^26^.

There was a decrease of − 41.4% in the total visits at UHS for the Fiscal Year (AY) 2020–2021 when compared to AY 2018–2019. For medical division that decrease was -50.8% and for mental health Division − 15.3% for that same year^27, 28^.

### Operational definitions

We defined psychiatric medication prescriptions as the presence of any of the following psychiatric medications: alprazolam, bupropion hcl er (sr), bupropion hcl er (xl), buspirone hcl, citalopram hydrobromide, clonazepam, clonidine hcl, cymbalta, duloxetine hcl, effexor xr, escitalopram oxalate, fluoxetine hcl, gabapentin, hydroxyzine hcl, klonopin, lamictal, lamotrigine, lexapro, lithium carbonate er, lorazepam, mirtazapine, paroxetine hcl, paxil, propranolol hcl, propranolol hcl er, prozac, sertraline hcl, trazodone hcl, venlafaxine hcl er, wellbutrin sr, wellbutrin xl, xanax, and zoloft.

### Sample

Psychiatric medication prescriptions written at UHS during medical visits (primary care, sexual health, gynecology, travel, and psychiatry) were included during the analyzed period.

### Proposed analysis

The proposed analysis is descriptive study with a cross-sectional and an ecological component from a retrospective cohort of college students that received care services in UHS. The study includes an observed to expected analysis of the prescribed psychiatric medications in 2020 and in 2021, compared to historical data and a time series analysis of monthly psychiatric medication prescriptions since 2015. We include both approaches in order to show not only the excess of psychiatric medication prescriptions during the global health crisis, but also to disclose the steady increase those prescriptions had over the years in a college setting.

#### Average prescriptions per analyzed period

First, described the average number of psychiatric medication prescriptions at the university clinic from January 1, 2015, to December 31, 2019, and during 2020 and 2021 separately.

#### Observed vs expected psychiatric medication prescriptions analysis

Then, we assessed the excess of psychiatric medication prescriptions, using the observed psychiatric medication prescriptions in medical services in 2020 and in 2021 separately and the expected psychiatric medication prescriptions in medical services to have occurred on a monthly basis in the 2015–2019 period (based on historical data). We compared monthly observed psychiatric medication prescriptions for 2020 and for 2021 with the baseline average number of prescriptions per month and the higher limit of the 95% confidence interval (CI 95%) derived from the historic average from 2015- 2019. We developed this descriptive analysis for the whole sample and per sex. We then obtained the p-value for hypothesis testing the null hypothesis: 2020, and 2021 psychiatric medication prescriptions don´t differ from the estimated prescriptions in 2015–2019, and the alternative hypothesis: 2020, and 2021 psychiatric medication prescriptions are different from the estimated psychiatric medication prescriptions in 2015–2019. Statistical significance for rejecting the null hypothesis was declared when the p-value was < 0.05. The excess of prescriptions was described as percentage.

#### Time series analysis

Finally, we developed a retrospective time series analysis of psychiatric medication prescriptions prescribed in medical departments. Including the three terms of enrollment that cover the calendar year, Fall, Spring and Summer, there is an average population of 54,500 enrolled students per year and about 5000 medical visits per month. Data were obtained from the electronic health records through PnC (Healthcare software). The analysis includes psychiatric medication prescriptions in medical visits per month for the whole sample and stratified by biological sex. For the analysis per sex, visits and prescriptions with biological sex labeled as “unknown” were removed from the analysis by sex but not from the whole sample analysis.

For the analysis of the time series, we developed the decomposition of the series into its components: trend, seasonality and residual or reminder, applying “Seasonal and Trend decomposition using locally estimated scatterplot smoothing or LOESS (STL)”^29^. This is a method for fitting a smooth curve between two variables or fitting a smooth surface between an outcome and up to four predictor variables methods. The overall level is removed from the seasonal component and added to the trend component. This process is iterated a few times. The remainder component is the residual from the seasonal plus trend fit^30^. We worked with the R program and its statistical packages "stats", "forecast", "seatests."

##### Seasonality

We assessed seasonality through different techniques of visualization and specific statistical tests (Ollech-Webel overall seasonality test, WO). The series was considered to have seasonality when the p-value for the modified QS test was ≤ 0.01; or the p-value for the modified QS test was > 0.011 and the p-value for the modified Friedman test was ≤ 0.002. In other words, if the p-value of the QS-test is below 0.01 or the p-value of the kw-test is below 0.002, the WO-test will classify the corresponding time series as seasonal^31^.

##### Trend

We analyzed the visualization of the trend from 2015 to 2021. We also estimate the trend of the line and its inclination with the number of prescriptions of the periods before the pandemic (2015–2019) and after the pandemic (2021). Due to the fact that 2020 was an atypical year in terms of primary care health services, we decided to remove this year from the slope analysis of the trends. To assess whether cases increased, decreased, or remained the same before (BP) and after (AP) the pandemic, data from the decomposition of the time series were used to calculate the slope of the line. Considering that the slope of a line is equal to the vertical displacement between two points divided by the horizontal displacement between those same two points, that is:

P1(x1, y1) P2(x2, y2).

The formula for the slope of a line:

Being:

x1, y1 the value of the month of January 2015 for the BP period and 2021 for the AP.

x2, y2 the value of the month of December 2019 for the BP period and 2021 for the AP.

According to the result of m, a trend was considered:

- Increasing when the value was greater than 0,
- Decreasing when m was less than 0.
- No trend when m was equal to 0.

##### Residual or remainders

This is the random component that remains after removing the trend and seasonality of the original time series. They were plotted and visually analyzed.

## Results

### Descriptive analysis

**Figure 1:**
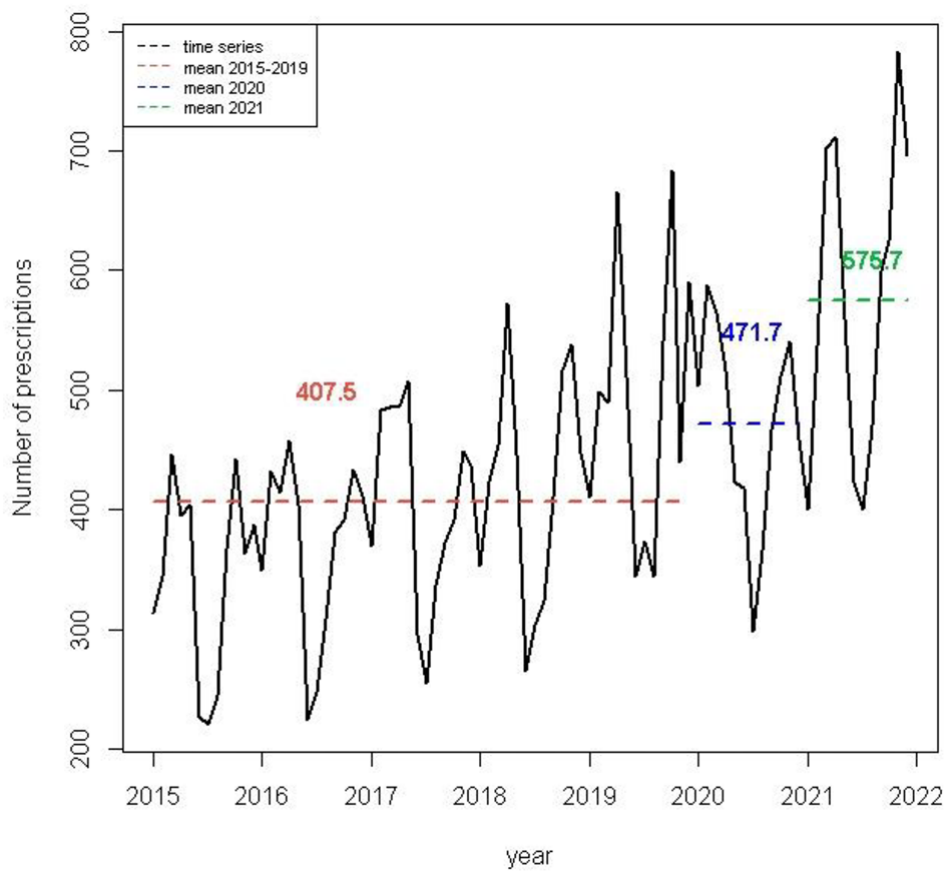
Mean psychiatric medication prescriptions in medical Services. UHS, UW-Madison, 2015–2021.

In the seven years analyzed, there were 36,017 prescriptions of psychiatric medication medications done by the included medical departments. From 2015 to 2019 the mean per month was 407.5 prescriptions; during 2020 the monthly average was 471.7 and during 2021 the mean of prescriptions was higher, 575.7 prescriptions per month (Fig. 1). The average historical 2015–2019 for the chosen medical visits was 50,394 and the number of medical visits for 2020 and 2021 was 31,754 and 46,670 respectively.

### Excess of psychiatric medication prescriptions

Table 1 shows average monthly psychiatric medication prescriptions in 2015–2019 and monthly prescriptions in 2020 and in 2021. For the whole sample in 2020 the annual percentage increase of psychiatric medication prescriptions from the historical baseline average was 15.8% and 3.5% when compared to the 95% CI upper limit (p value: 0.012). All months but April, May, July, September, and December had a significant increase. The month with the highest increase was June with a 54% increase from baseline average and 32.6% increase when compared to the 95% CI upper limit (p value: 0.000). When stratifying by sex in 2020, the increase of psychiatric medication prescriptions from historical baseline average for females was 21.3% and 4.6% when compared to the 95% CI upper limit (p value: 0.004). In males, 2020 showed a non-significant percentage decrease of psychiatric medication prescriptions from the historical average, − 5.3% and − 10.9% when compared to the 95% CI upper limit (p value: 0.537).

**Table 1:** Excess of psychiatric medication prescriptions in Medical Services and its historical comparison (2015–2019).

	Month	2015–2019 Monthly average prescriptions (95% CI)	2020 Psychiatric Medication Prescriptions	2020% above baseline	2020% above threshold	p value	2021 Psychiatric Medication Prescriptions	2021% above baseline	2021% above threshold	p- value
Total	January	360 (329–391)	504	40.1	29.0	0.000	400	11.2	2.4	0.035
	February	437 (384–490)	588	34.6	20.0	0.000	537	22.9	9.6	0.000
	March	458 (431–485)	565	23.3	16.4	0.000	703	53.4	44.9	0.000
	April	516 (423–608)	517	0.2	− 15.0	0.958	711	37.8	16.9	0.000
	May	455 (409–501)	424	− 6.8	− 15.4	0.148	560	23.1	11.8	0.000
	June	271 (227–315)	418	54.0	32.6	0.000	423	55.9	34.2	0.000
	July	280 (228–333)	299	6.6	− 10.3	0.266	400	42.7	20.0	0.000
	August	312 (277–347)	366	17.4	5.6	0.002	471	51.1	35.9	0.000
	September	415 (352–478)	467	12.5	− 2.4	0.11	599	44.3	25.2	0.000
	October	485 (379–592)	512	5.5	− 13.5	0.223	625	28.8	5.6	0.000
	November	445 (391–499)	541	21.6	8.3	0.000	783	76.0	56.8	0.000
	December	455 (386–525)	459	0.8	− 12.6	0.866	696	52.8	32.6	0.000
	Annual Average	407 (359–456)	472	15.8	3.5	0.012	576	41.3	26.3	0.000
Female	January	240 (209–270)	358	49.4	32.4	0.000	299	24.8	10.6	0.001
	February	288 (260–317)	414	43.6	30.7	0.000	375	30.0	18.4	0.000
	March	304 (276–331)	396	30.3	19.6	0.000	501	64.9	51.3	0.000
	April	344 (278–411)	370	7.4	− 10.0	0.167	547	58.8	33.1	0.000
	May	298 (260–335)	296	− 0.5	− 11.6	0.926	393	32.1	17.4	0.000
	June	176 (143–209)	288	63.3	37.5	0.000	319	80.8	52.3	0.000
	July	190 (150–230)	209	10.0	− 9.1	0.168	297	56.3	29.2	0.000
	August	200 (169–230)	266	33.3	15.6	0.000	347	73.8	50.8	0.000
	September	280 (224–337)	340	21.3	0.8	0.003	441	57.3	30.8	0.000
	October	322 (236–408)	324	0.7	− 20.5	0.902	472	46.7	15.8	0.000
	November	320 (247–393)	381	19.1	− 3.0	0.006	590	84.4	50.3	0.000
	December	314 (253–374)	332	5.9	− 11.2	0.298	498	58.8	33.3	0.000
	Annual Average	273 (229–317)	331	21.3	4.6	0.004	423	55.1	33.7	0.000
Male	January	125 (114–136)	140	12.2	3.0	0.173	93	-25.5	− 31.6	0.044
	February	150 (125–175)	168	12.0	− 3.9	0.141	150	0.0	− 14.2	1.000
	March	153 (143–163)	156	1.7	− 4.5	0.833	182	18.6	11.4	0.021
	April	169 (139–198)	140	− 17.0	− 29.3	0.027	151	− 10.4	− 23.7	0.175
	May	155 (146–164)	122	− 21.4	− 25.7	0.077	151	− 2.7	− 8.1	0.736
	June	93 (79–108)	122	30.9	13.2	0.028	96	3.0	− 11.0	0.771
	July	89 (77–101)	86	− 3.4	− 14.9	0.751	93	4.5	− 8.0	0.671
	August	110 (99–121)	90	− 18.3	− 25.7	0.054	105	− 4.7	− 13.4	0.620
	September	130 (122–138)	120	− 7.8	− 13.3	0.371	137	5.2	− 1.1	0.551
	October	160 (141–178)	130	− 18.5	− 26.9	0.019	136	− 14.8	− 23.5	0.061
	November	152 (141–163)	147	− 3.4	− 10.0	0.177	173	13.7	5.9	0.091
	December	138 (121–155)	117	− 15.0	− 24.3	0.791	171	24.3	10.6	0.044
	Annual average	135 (127–144)	128	− 5.3	− 10.9	0.537	136.5	0.9	− 5.1	0.92

Total and monthly prescriptions for The whole sample and per sex. UHS, UW-Madison 2020 and 2021.

For the whole sample in 2021, the annual percentage increase of psychiatric medication prescriptions from the historical baseline average was 41.3% and 26.3% when compared to the 95% CI upper limit (p value: 0.000). All months were significant for this increase. The month with the highest increase was November with an increase from baseline average of 76% and 56.8% when compared to the 95% CI upper limit (p value: 0.000). When stratifying by sex, females also show total and monthly increase of psychiatric medication prescriptions to be significant when compared to 2015–2019 (55.1%, 33.7%; p value: 0.000). In males, 2021 showed a non-significant percentage increase of psychiatric medication prescriptions from historical, 0.9% and -5.1% when compared to the 95% CI upper limit (p value: 0.920).

### Time series analysis

**Figure 2:**
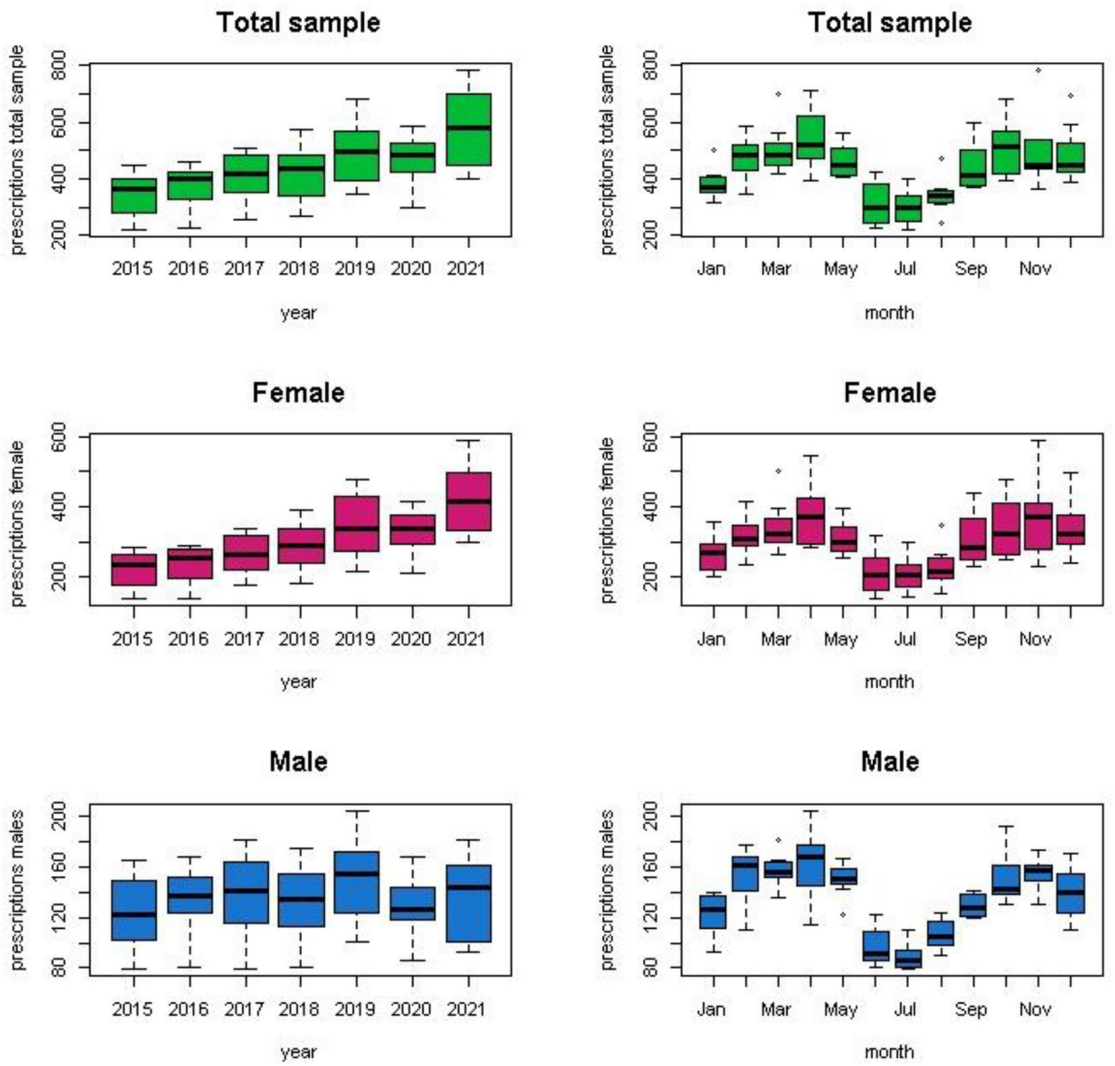
Psychiatric medication prescriptions in medical services per year and monthly. Whole sample and per sex. UHS, UW- Madison. 2015–2021.

**Figure 3:**
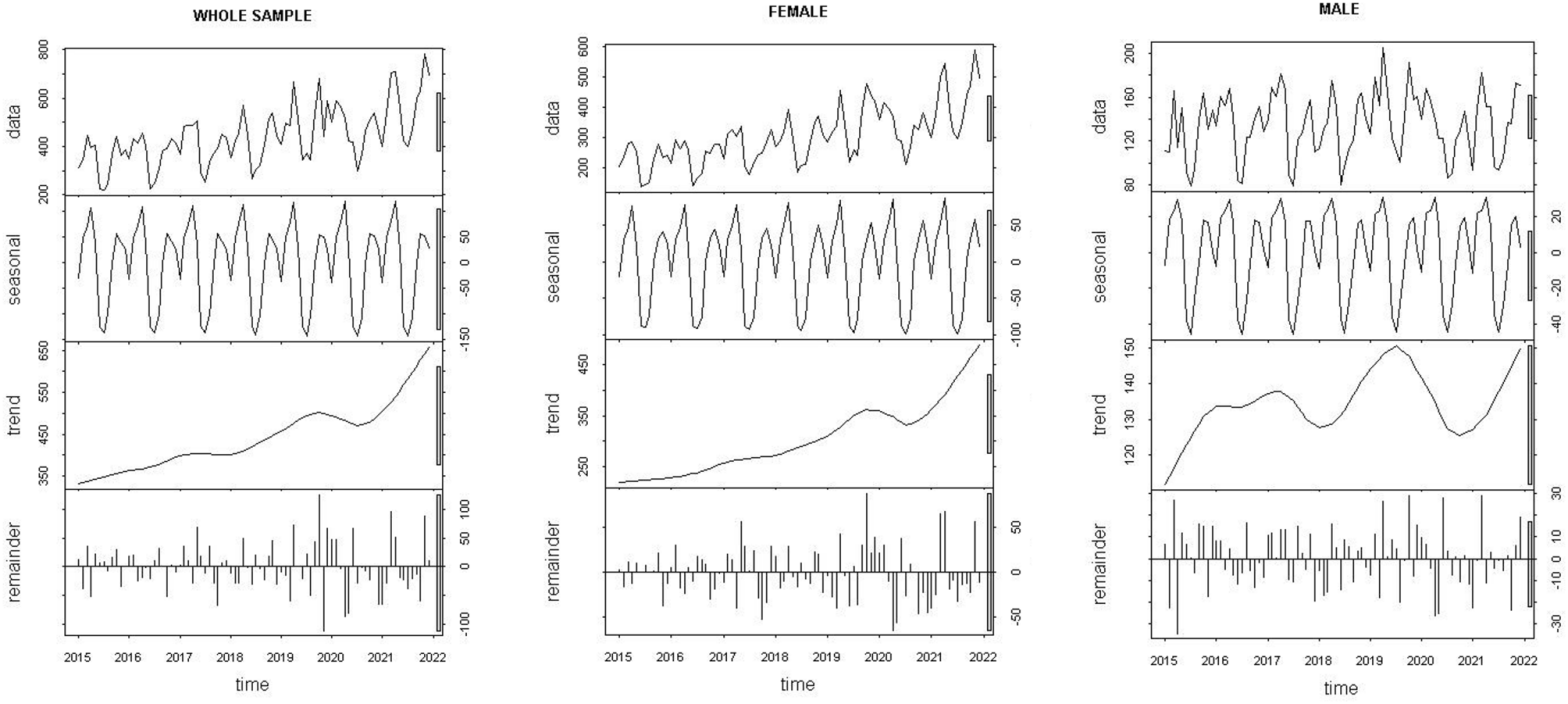
Decomposition of the additive Time Series. Psychiatric Medication Prescriptions in medical services for the whole sample and per sex. UHS, UW-Madison, 2015–2021.

When analyzing the time series of psychiatric medication prescriptions in medical services, we see different means before, during and after the pandemic. Figure 2 shows the annual and monthly pattern of the prescriptions; annual prescriptions increased over the years and in the monthly analysis we see peaks of prescriptions in April/May and October/December for the whole sample and both for females and males. Figure 3 shows the decomposition of the time series into its components for the whole sample, females, and males. When breaking down the time series of “number of psychiatric medication prescriptions” we observe in each of its components a clear upward trend and seasonality. We detect seasonality for the whole sample and per sex.

#### Seasonality

The decomposition of the time series into its components shows a clear seasonality that was significant when considering the whole sample and both women and men. The Webel-Ollech general seasonality test (WO test) confirms this seasonality by classifying the health prescriptions (P-value QS-test = 8.74 e^−7^ and P-value kw-test = 2.92 e^−6^). When analyzing seasonality by sex, we find a similar pattern in both sexes. There is a gradual increase in psychiatric medication prescriptions observed from April to May, then a decrease in June and July, and increasing again in September through December. This is significant both in women (women P-value QS-test = 5.38 e^−9^, P-value kw-test = 1.74 e^−6^ and in men (men P-value QS-test = 2.4 e^−7^, P-value kw-test = 9.55 e^−5^).

#### Trend

Figure 3 shows an increasing trend in the number of prescriptions observed in the general population and per sex. After eliminating the seasonality of the time series, we observed a growing trend in psychiatric medication prescriptions from January 2015 to December 2019, a decrease in 2020 and a new increment in 2021. When relating the trend pattern with the residuals, we observe a unique pattern in 2020, during the time when visits were reduced. To assess the increase of psychiatric medication prescriptions, we analyzed the trend of two straight lines before pandemic (BP) and after pandemic (AP). The slope of the line was calculated between January 2015 and December 2019 (BP) and then from January 2021 to December 2021.Data shows an increasing trend in the number of prescriptions observed in the general population with a BP slope of 4.7 (angle 77°) and AP at 26.9 (angle 87°). When broken down by sex, a similar behavior was observed in women, going from a slope of 3.7 AP (angle 74°) to 18.1 AP (angle 86°); in men, although they had fewer prescriptions, the upward trend was more marked, observing an BP slope of 0.8 (angle 0.4°) and AP of 7.1 (angle 81°).

#### Residual or remainders

After removing the seasonality and the trend from the time series, we observe that in 2020 the residuals showed changes that could not be attributed to neither the trend nor the seasonality. This could be related to the fact that UHS reduced medical visits to its minimum during 2020.

## Discussion

Replicating previous work, we found that psychiatric medication prescriptions for college students have continued to rise, and the chronic stress caused by the COVID pandemic appeared to accelerate these numbers with the number of prescriptions rising on average from 407 in the period 2025–2019 to 576 in 2021 with a 41.3% overall increase. These findings align with the literature that shows a significant increase in the use of psychiatric medications by college students in the past 10 years, prior to pandemic, where anti-anxiety prescriptions increased from 3% to 7.6% and antidepressants prescriptions went from 8% to 15.3%, to name^1, 32^. This overall increase in psychiatric medication prescriptions has been particularly high among adolescents (ages 13–19) in the general population^33, 34^.

When we examined the observed vs expected number of psychiatric medication prescriptions by year, we found a 16% increase in 2020, compared to the average in 2015–2019 and an astonishing 42% more prescriptions in 2021 compared to the average 2015–2019. The general increase in psychiatric medication use likely reflects the growing rates of psychiatric problems on campus. The doubling of antidepressant and anti-anxiety use is not surprising, given the growing rates of depression and anxiety^32^.

While these increases were in line with our hypotheses that the number of psychiatric medication prescriptions would increase as a result of the stress caused by the pandemic, these numbers exceeded our expectations. Though data from point-in-time natural disasters have suggested a return to baseline levels of pharmacological intervention after a few months^10^ it is not known what shift in prescription practices will occur over the course of a prolonged global pandemic. It is possible that we need more time to adapt to a “post-pandemic” world to adjust to baseline levels^35, 36^. However, it is also possible that a new baseline of higher prescriptions will be established, which has implications for future staffing of a medical center or increased focus on university programs that can teach university students coping skills on a broader scale.

In addition to having the proper number of staff to provide care for college students with mental health needs, our results suggest it is also important to be aware that these needs change with the academic year. We found increases in psychiatric medication prescriptions from September to December, corresponding to academic Fall terms of all the analyzed years, and again from April to May that are the final months of the academic Spring terms for all analyzed years^37^. These findings replicated what others have found^6, 38^. It is possible that these prescriptions are related to academic demands (e.g., finals time, graduation)^39, 40^. Of note, we have these trends in the academic seasonality for prescriptions for both men and women in our sample, but the observed vs expected analysis showed a higher increase only for females. This discrepancy could be related to a sustained increase in psychiatric medication prescriptions both for male and female prior to pandemic^1^ and a steeper increase in the prescriptions in female students during pandemic months. In line with this, several studies show a higher level of distress in female college students during the pandemic^41, 42^. Locally, published data shows that the average PHQ-9 increased for both male and female during the Fall term 2020, but this increase was higher among women^43^. Differences in rates of psychiatric medication between men and women were seen in the general population during the pandemic, with women reporting higher rates of anxiety and depression at least due in part to increased childcare demands, which may be a factor for our sample as well^44, 45^.

We do have a number of limitations to our study. First, we used a cross- sectional and an ecological approach, therefore no directionality or association can be established. Our sample includes only college students, so it may not be generalizable to non-college populations. We were limited to binary sex analysis by sourcing through registrar data, which does not reflect the nuance of gender identity and the role it may factor into these results. We did not include other demographics such as college year, due to limited access to PHI. In addition to our methodology, the clinic we studied used a triage process for medical visits. During 2020, the number of medical visits at UHS were reduced to its minimum. UHS data shows that for Fiscal Year 2020–2021 (July 2020-June 2021), the total number of medical visits decreased over − 49% compared to historical data (average for Fiscal years 2016–2017 to 2019–2020) and a decrease in medical visits of almost -51% when compared to Fiscal Year 2018–2019^27, 28, 46, 47^. This could have reduced the number of prescriptions in this period, making the approach of comparing the number of prescriptions a conservative one. This is reflected in the decrease in the trend in 2020 that could be explained not only with the triaging but also because many students moved back to their home residences during the online courses occurred during 2020. An additional limitation is that our results are within a specific window of time when factors known to increase depression and anxiety such as social isolation were uniquely high; as such, these results may be different in future windows when social interaction increases.

Overall, we found that psychiatric medication prescriptions have continued to rise through the years, with a large increase occurring during the pandemic months. In addition, we found that these increases reflect the academic year, which is important for university health centers to consider when they are planning to staff clinics, introduce programs, and plan for the best way to treat college students with psychiatric medication difficulties in the future.

## Data Availability

The datasets used and/or analyzed during the current study available from the corresponding author on reasonable request.
